# Supplementary material for: The hinge-engineered IgG1-IgG3 hybrid subclass IgGh47 potently enhances Fc-mediated function of anti-streptococcal and SARS-CoV-2 antibodies
Source: Nat Commun. 2024 Apr 27;15:3600. doi: 10.1038/s41467-024-47928-8 (PMC11055898; doi:10.1038/s41467-024-47928-8)
Supplement: Supplementary file 1 — Supplementary Information [file 41467_2024_47928_MOESM1_ESM.pdf]

The hinge-engineered IgG1-IgG3 hybrid subclass IgGh$_{47}$ potently enhances Fc-mediated function of anti-streptococcal and SARS-CoV-2 antibodies

*Arman Izadi[1], Yasaman Karami[2,3], Eleni Bratanis[1], Sebastian Wrighton[1], Hamed Khakzad[2], Maria Nyblom[4], Berit Olofsson[1], Lotta Happonen[1], Di Tang[1], Martin Sundwall[1], Magdalena Godzwon[5], Yashuan Chao[1], Alejandro Gomez Toledo[1], Tobias Schmidt[6], Mats Ohlin[5], Michael Nilges[3], Johan Malmström[1], Wael Bahnan[1], Oonagh Shannon[1,7], Lars Malmström[1], and Pontus Nordenfelt[1,8]**

[1] *Department of Clinical Sciences Lund, Infection Medicine, Faculty of Medicine, Lund University, Lund, Sweden*

[2] *Université de Lorraine, CNRS, Inria, LORIA, F-54000, Nancy, France*

[3] *Institut Pasteur, Université Paris cite, CNRS UMR3528, Structural Bioinformatics Unit, Department of Structural Biology and Chemistry, F-75015, Paris, France*

[4] *Department of Biology & Lund Protein Production Platform (LP3), Lund University, Lund, Sweden*

[5] *Department of Immunotechnology and SciLifeLab Drug Discovery and Development Platform, Lund University, Lund, Sweden*

[6] *Department of Clinical Sciences Lund, Division of Pediatrics, Faculty of Medicine, Lund University, Lund, Sweden*

[7] *Section for Oral Biology and Pathology, Faculty of Odontology, Malmö University, Malmö, Sweden*

[8] *Department of Laboratory Medicine, Clinical Microbiology, Skåne University Hospital Lund, Lund University, Lund, Sweden*

**correspondence to pontus.nordenfelt@med.lu.se*

Supplementary Materials

Supp. Table 1. Crystallography data table over data used in structure determination.

Supp. Table 2. Data summary from MD simulations.

Supp. Fig. 1. Flow cytometry-based gating strategies and control experiments.

Supp. Fig. 2. Flow cytometry-based gating strategies for monocytes.

Supp. Fig. 3. Flow cytometry-based gating strategies for neutrophils.

Supp. Fig. 4. The root mean square deviations and fluctuations for M1-IgGs.

Supp. Fig. 5. Changes of angles along the MD simulations for M1-IgG1, M1-IgG3, and M1-IgGh$_{62}$ systems.

Supp. Fig. 6. Clustering of MD confirmations for IgG1-M1, IgG3-M1, and IgGh$_{62}$-M1 systems.

Supp. Fig. 7. Binding energy estimated using FoldX.

Supp. Fig. 8. Computational alanine scanning of the Fab1 residues at the interface with the M1.

Supp. Fig. 9. Computational alanine scanning of the Fab2 residues at the interface with the M1.

Supp. Fig. 10. Gating strategy and directly labeled affinity measurements.

Supp. Fig. 11. Binding characteristics of human IgGh47, IgG1, and IgG3 to Fc receptors as determined by surface plasmon resonance.

Supp. Fig. 12. Gating strategy for THP-1 cell phagocytosis of spike-beads.

Supp. Fig. 13. Integrative structural modeling of M1-IgG3.

Supp. Fig. 14. Ab25 crystal structure.

Supplementary Table 1

Crystallography data table over data used in structure determination. Data in parenthesis correspond to the highest resolution shell.

Data collection	Ab25
Space group	P 1 21 1
Cell dimensions<br>a, b, c (Å)	86.90 71.89 146.01
α, β, γ (°)	90.00 90.10 90.00
Wavelength (Å)	0.97625
Resolution (Å)	48.67-1.88 (1.95-1.88)
Rmerge	0.119 (1.107)
Number of observations	1015596 (96059)
Number of unique observations	146332 (14268)
Mean I/σ(I)	11.7 (1.9)
Completeness (%)	99.9 (99.7)
Multiplicity	6.9 (6.7)
CC(1/2)	0.998 (0.835)
Refinement	
Resolution (Å)	44.152-1.880
R_{work}/R_{free}	0.2053/ 0.2550
Number of reflections	146123
R_{free} test set, reflections (%)	5.04
No. of non-H atoms	14174
R.m.s. deviation from ideal geometry	
bonds (Å)	0.008
angles (°)	1.089
Average B, all atoms (Å^2)	39.34 (range 13.63–113.89)
Ramachandran plot	
Favoured regions (%)	96.94
Allowed regions (%)	2.77
Outliers (%)	0.29
MolProbity clash score	4.79

Supplementary Table 2

Data summary from MD simulations.

Antibody	IgG1	IgG3	IgGh
**RMSD Fab 1 (Å)**	3.72 ±0.64	5.76 ±1.06	6.49 ±1.48
**RMSD Fab 2 (Å)**	4.89 ±0.89	5.16 ±0.77	4.43 ±0.8
**RMSD FC (Å)**	5.34 ±0.88	4.35 ±0.91	5.28 ±0.84
**RMSF Fab1 (Å)**	1.92 ±0.86	2.18 ±1.16	2.32 ±1.33
**RMSF Fab2 (Å)**	2.55 ±0.94	2.57 ±1.24	1.62 ±0.65
**RMSF FC (Å)**	1.64 ±1.01	1.63 ±0.96	2.88 ±1.68
**Fc Displacement (Å)**	94.10 ±6.77	111.01 ±24.50	167.13 ±17.60
**Fc-Fab angle °**	66° ±22°,	72° ±31°	31° ±14°
**Fc-M1 angle°**	92° ± 9°	86° ± 18°	93° ±14°
**Fab1-Fab2 angle°**	111°±15°	62°±8°	38° ±7°
**Fc-M1 angle span °**	39°	79°	66°

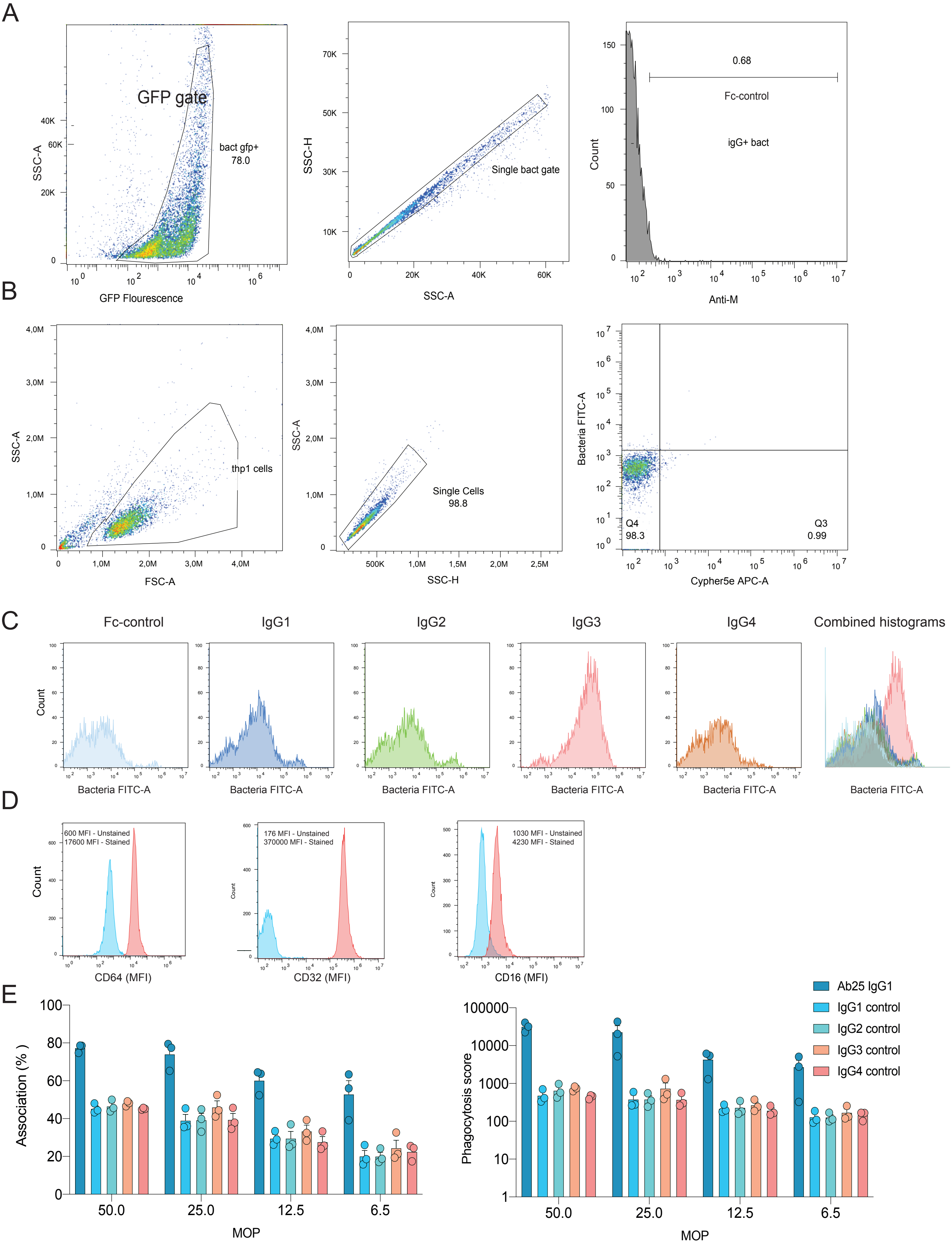

Supplemental Figure 1. Flow cytometry-based gating strategies and control experiments. A. Bacteria were gated for by green flourescence (GFP) and granularity, followed by a single cell gate. IgG reactivity was gated for by using a negative control. B. THP1 cells were gated for by size and granularity, then a single cell gate was made to exclude duplicate events followed by a internalization-association gate based on bacterial flourescence. C. Histograms showing engagement of THP1 cells with bacteria for different treatments. D. Fc-γ receptor profile of the THP1 cells. E. Subclass controls for the phagocytosis experiments, in E three indepedent experiments were performed (n=3).

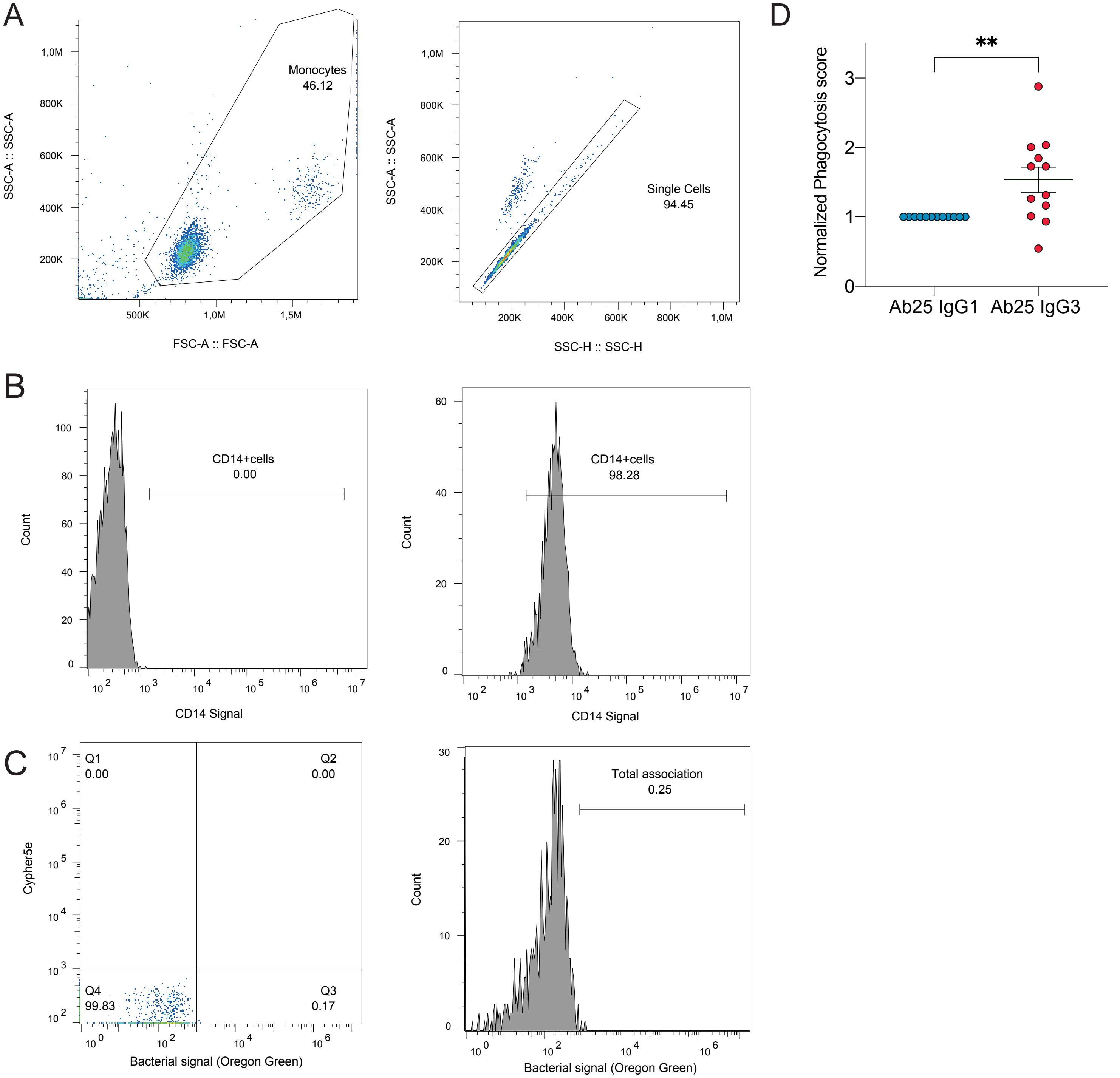

Supplementary Figure 2. Flow cytometry-based gating strategies for monocytes.
A Monocytes were gated for by size and granularity. Thereafter a single cell gate was done to eliminate duplicate events. **B** CD14 marker was thereafter used to gate for monocytes, first using unstained cells to set the gate. **C** To determine the level of internalization and association we used a negative control with cells only. Cells internalized with bacteria would be both positive for FITC (Oregon Green-stained bacteria) but also APC (Cypher5e pH-sensitive dye). Cells are associated with bacteria when only positive in the FITC-channel. A total association gate was set to include all bacteria being phagocytosed by the cells.
D Normalized phagocytosis score is shown, from 6 unique donors and 12 biologicaly independent experiments (each donor represented twice, N=12). Statistical assesment in D was done with two-tailed Mann-Whitney U test, were ** significes p-value below 0.01, ns is p-value > 0.05.

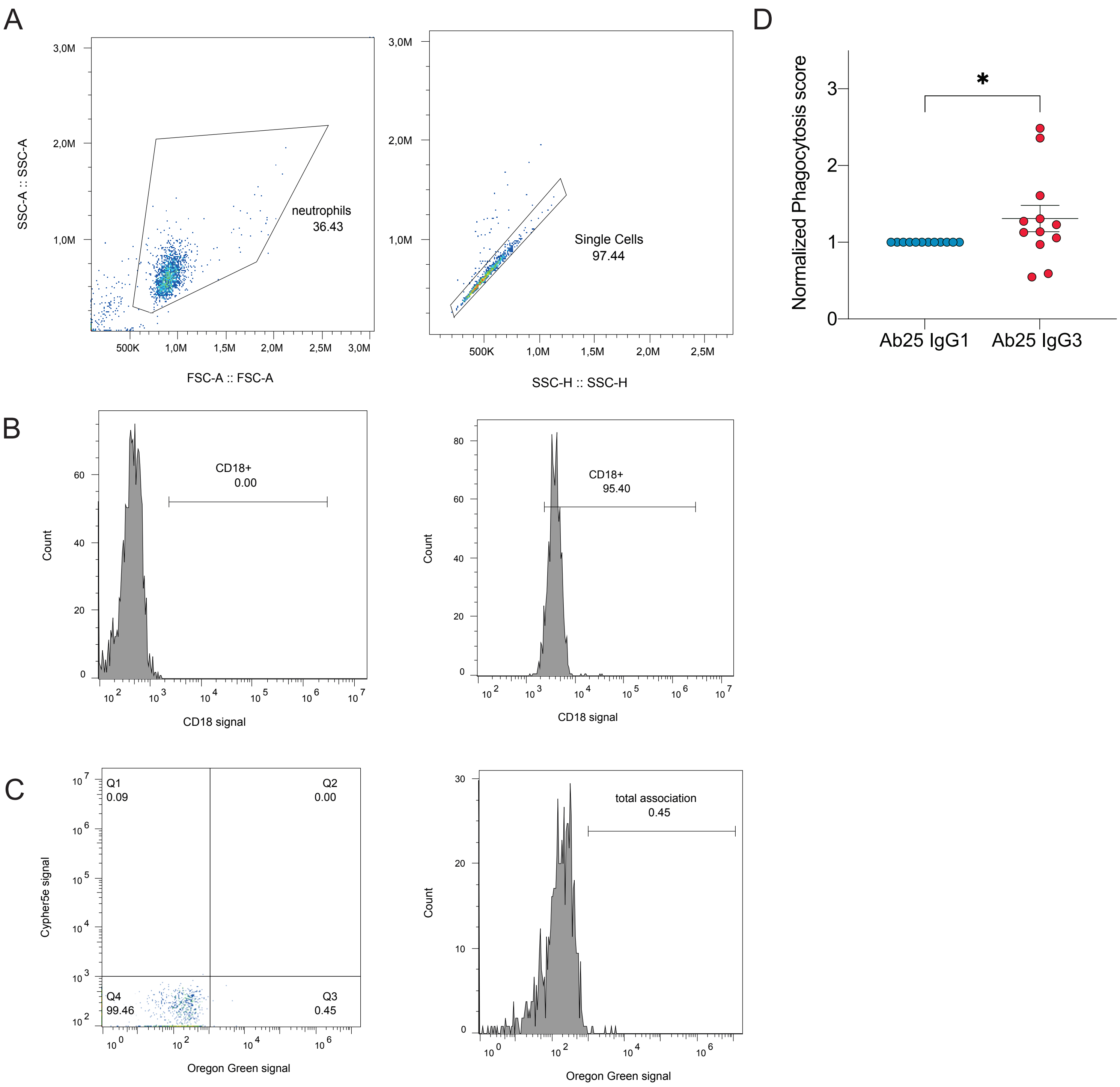

Supplementary Figure 3. Flow cytometry-based gating strategies for neutrophils.
A Neutrophils were gated for by size and granularity, then duplicate events were excluded by single cell gate. B An unstained control was used to set the CD18 gate to include CD18 positive neutrophils. C An internalization and association gate was set by using cells only from the CD18+gate. Similarly a total association gate was set. In D normalized phagocytosis score is shown, from 6 unique donors and 12 biologicaly independent experiments (each donor represented twice, n=12). Statistical assesment in D was done with two-tailed Mann-Whitney U test, were * significes p-value below 0.05 and above 0.05 is ns.

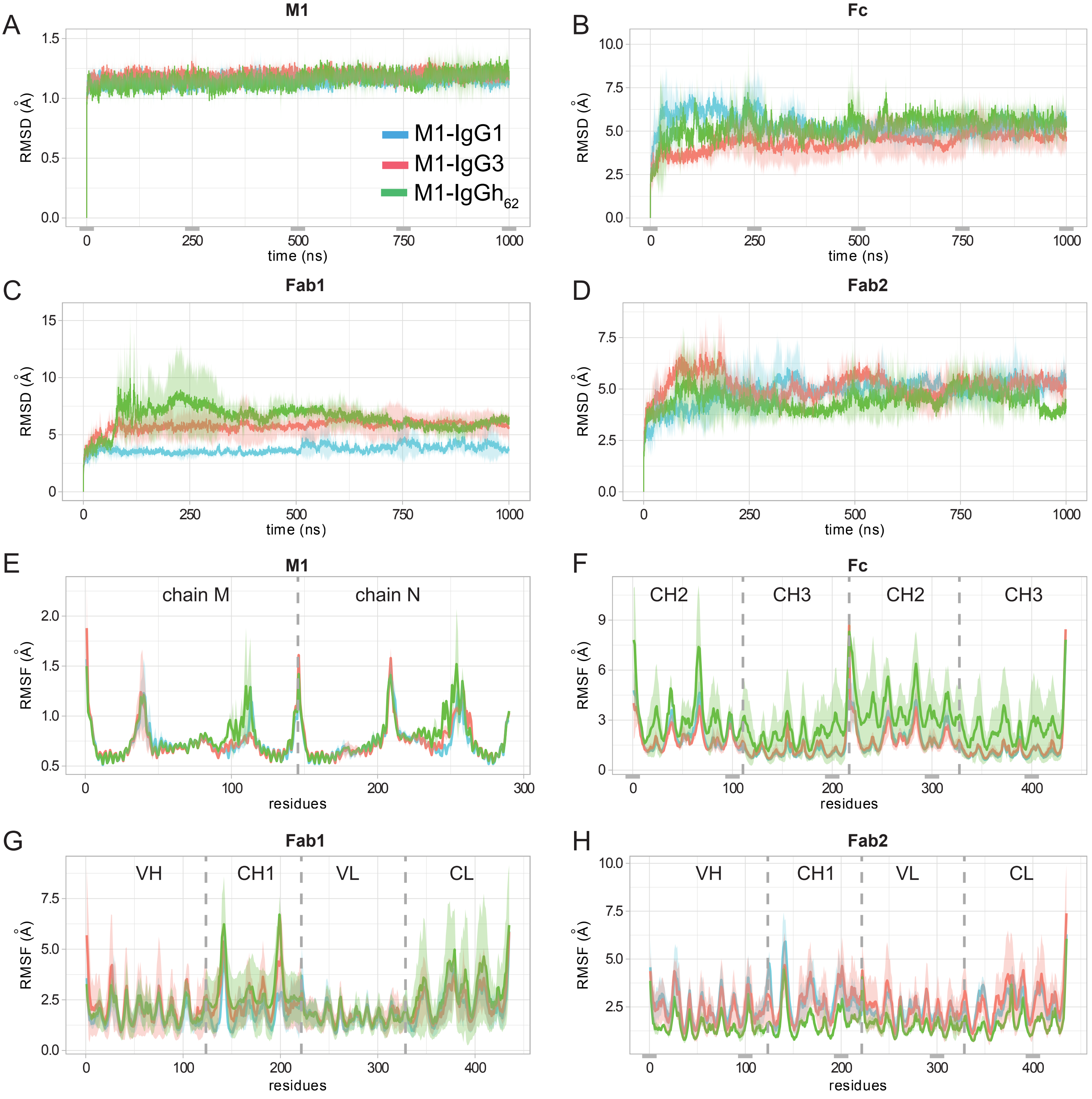

Supplementary Figure 4. The root mean square deviations and fluctuations for M1-IgGs. The RMSD from the equilibrated structure was computed on the C& atoms of each replicate and every domain: a) M1, b) Fc, c) Fab1, and d) Fab2. The RMSF was measured on the C& atoms with respect to the average conformation and averaged by residue, considering the last 900 ns of the MD simulations for the e) M1, f) Fc, g) Fab1, and h) Fab2 domains. The average values over the three replicates of each domain are reported in pink, cyan, and green for the M1-IgG1, M1-IgG3, and M1-IgGh systems, respectively. The shades correspond to the standard deviations.

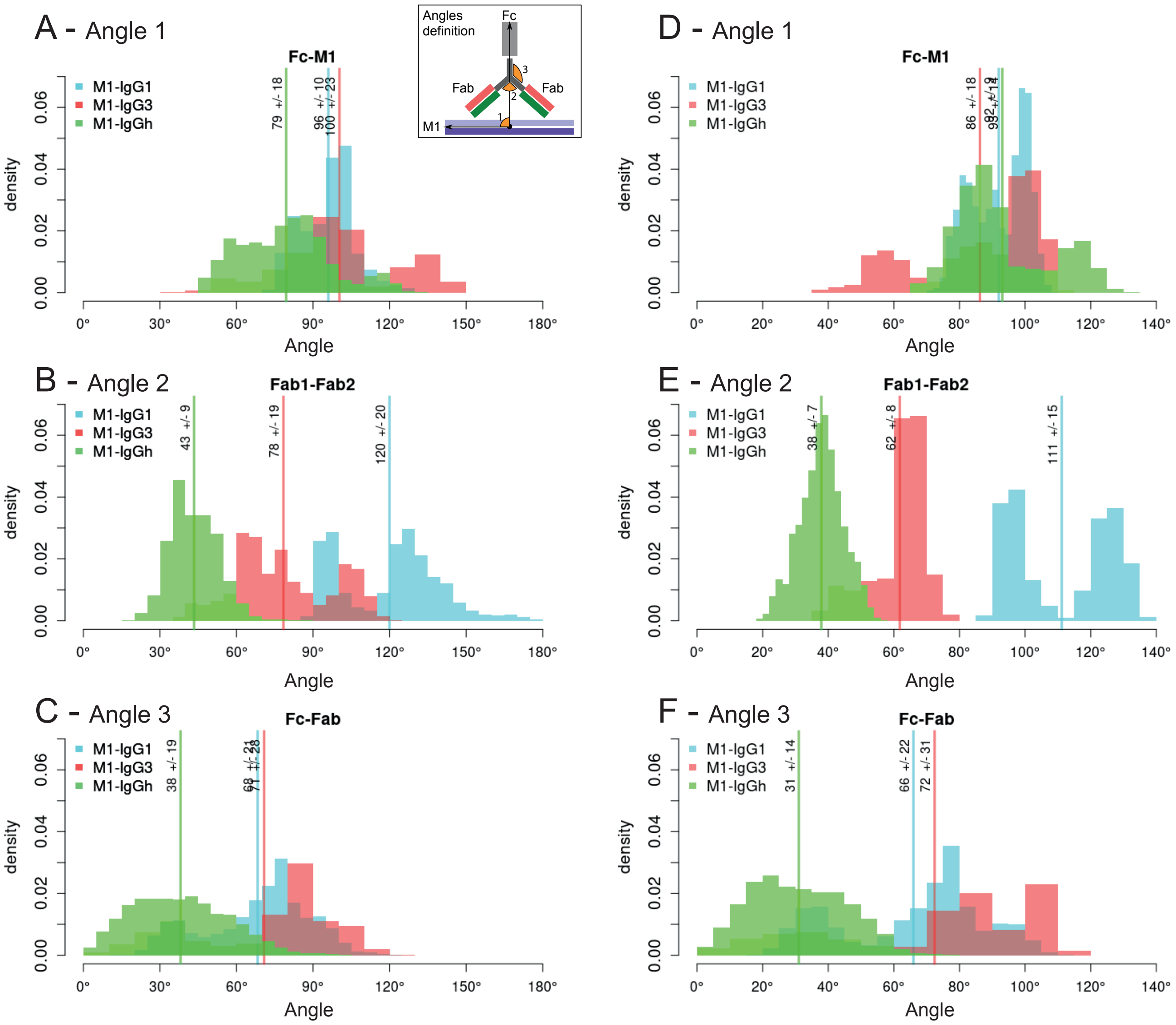

Supplementary Figure 5. Changes of angles along the MD simulations for M1-IgG1, M1-IgG3 and M1-IgGh$_{62}$ systems. The Y-axis shows the probability densities of the MD conformations for that specific angle while the X-axis shows the angle in degrees. A-C are the values reported over all the MD trajectories, while D-F are when the Fab domains are within 30 Å of the M1 protein. A and D show the angles between the Fc domain and M1. B and E show the angles formed between Fab 1 and Fab 2. C and F show the angles formed between the Fc and Fab domains. The IgG1-M1 system is highlighted in blue, red for IgG3-M1 and green for IgGh-M1 respectively.

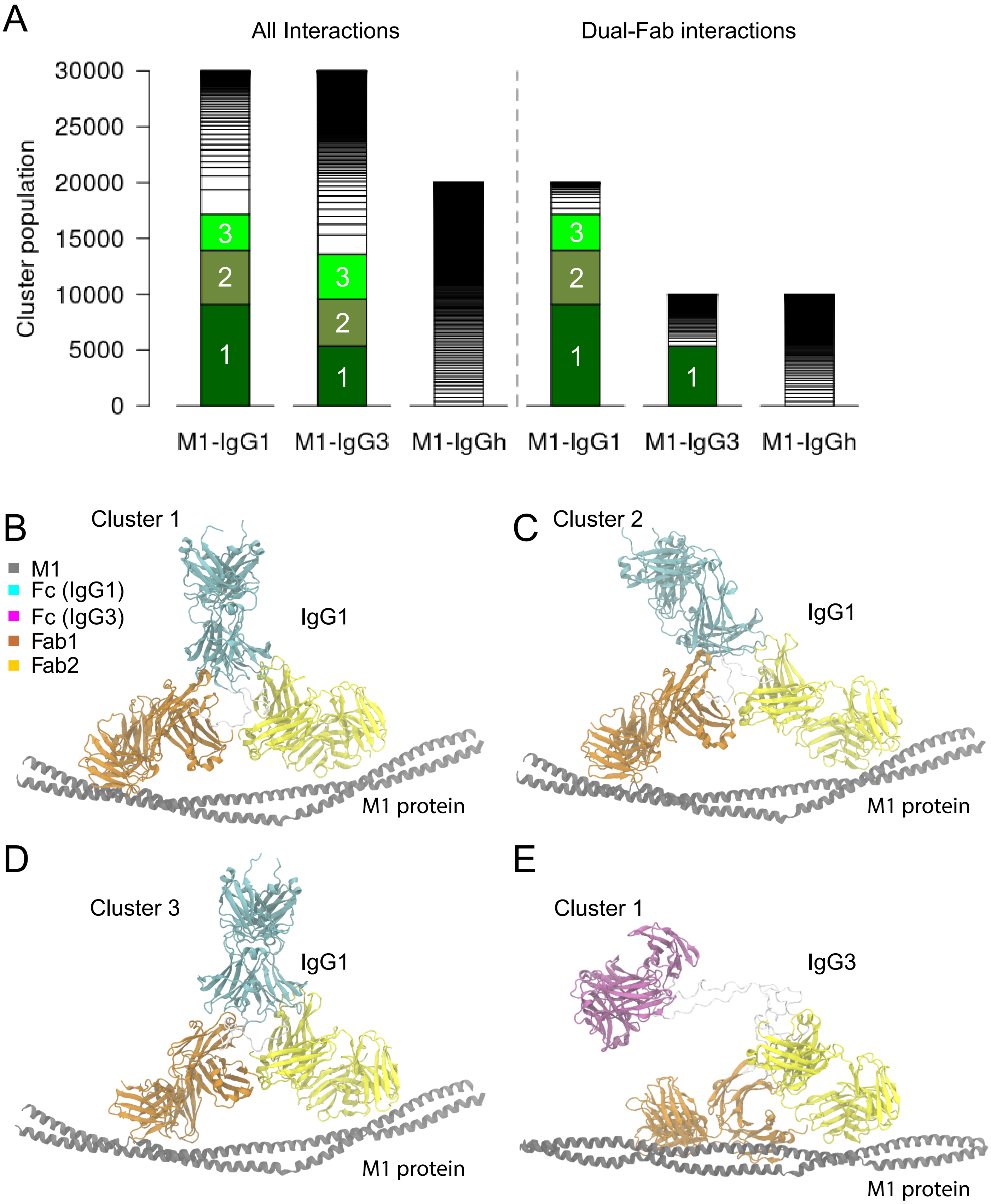

Supplementary Figure 6. Clustering of MD confirmations for IgG1-M1, IgG3-M1 and IgGh$_{62}$-M1 systems. A. The results are reported over all MD trajectories and only dual-Fab binding conformations. Highly populated clusters (with at least 3000 conformations) are colored in shades of green.The representative dual-Fab binding conformation for cluster 1 in B, cluster 2 in C and cluster 3 in D for M1-IgG1 and cluster 1 for M1-IgG3 in E.

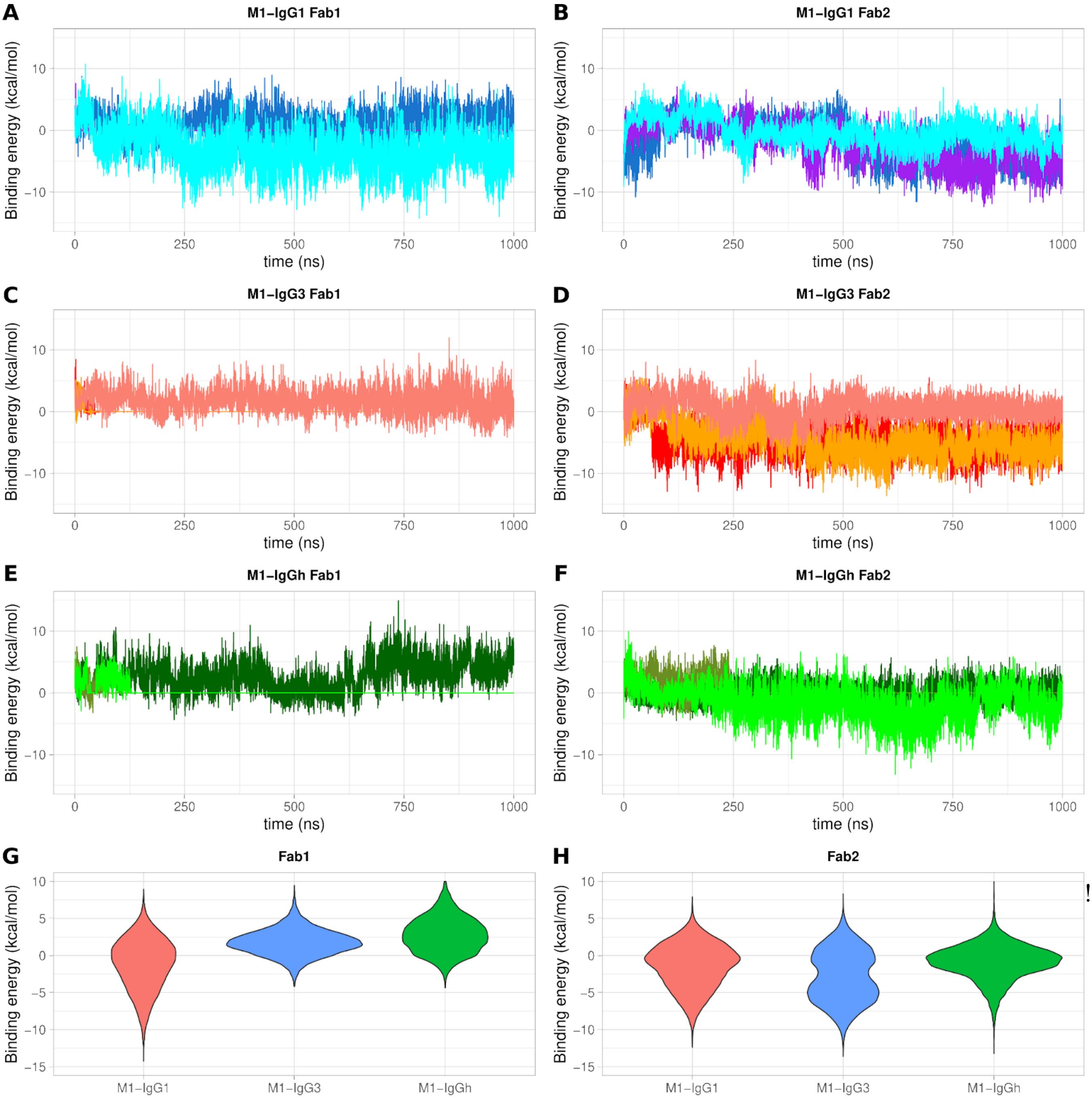

Supplemental Figure 7. Binding energy estimated using FoldX. The results are reported as time series for all the replicates of A-B) M1-IgG1 (shades of blue), C-D) M1-IgG3 (shades of red), and E-F) M1-IgGh$_{62}$ (shades of green), and as violin plot (G-H). The binding energy between the Fab1 domain and M1 protein are shown on the left and between the Fab2 and M1 protein on the right.

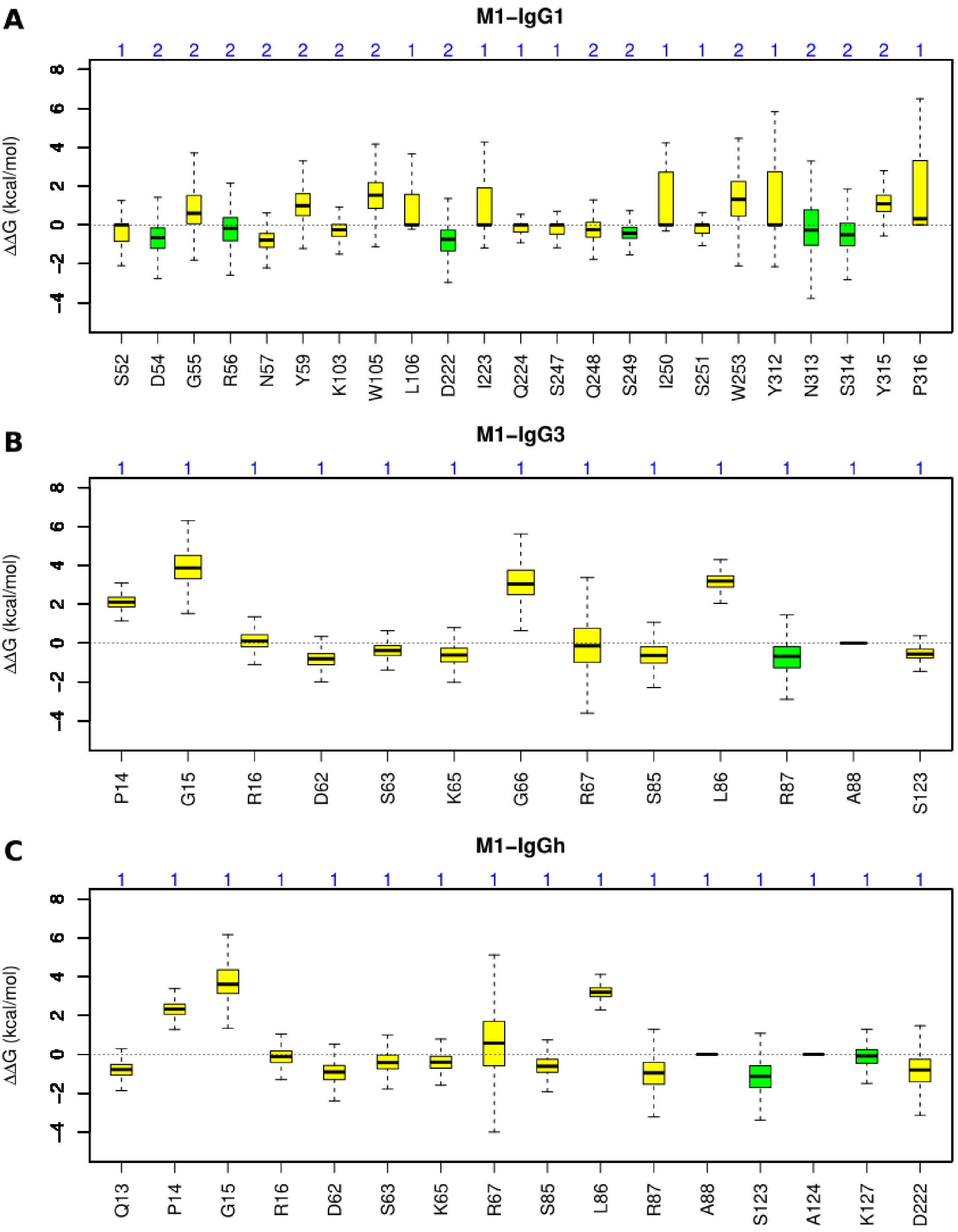

Supplemental Figure 8. Computational alanine scanning of the Fab1 residues at the interface with the M1. The results are reported for A) M1-IgG1, B) M1-IgG3, and C) M1-IgGh$_{62}$. The green boxes correspond to residues forming H-Bonds and salt bridges with the M1 protein. The blue nummer on top of each graph is the amount of replicates.

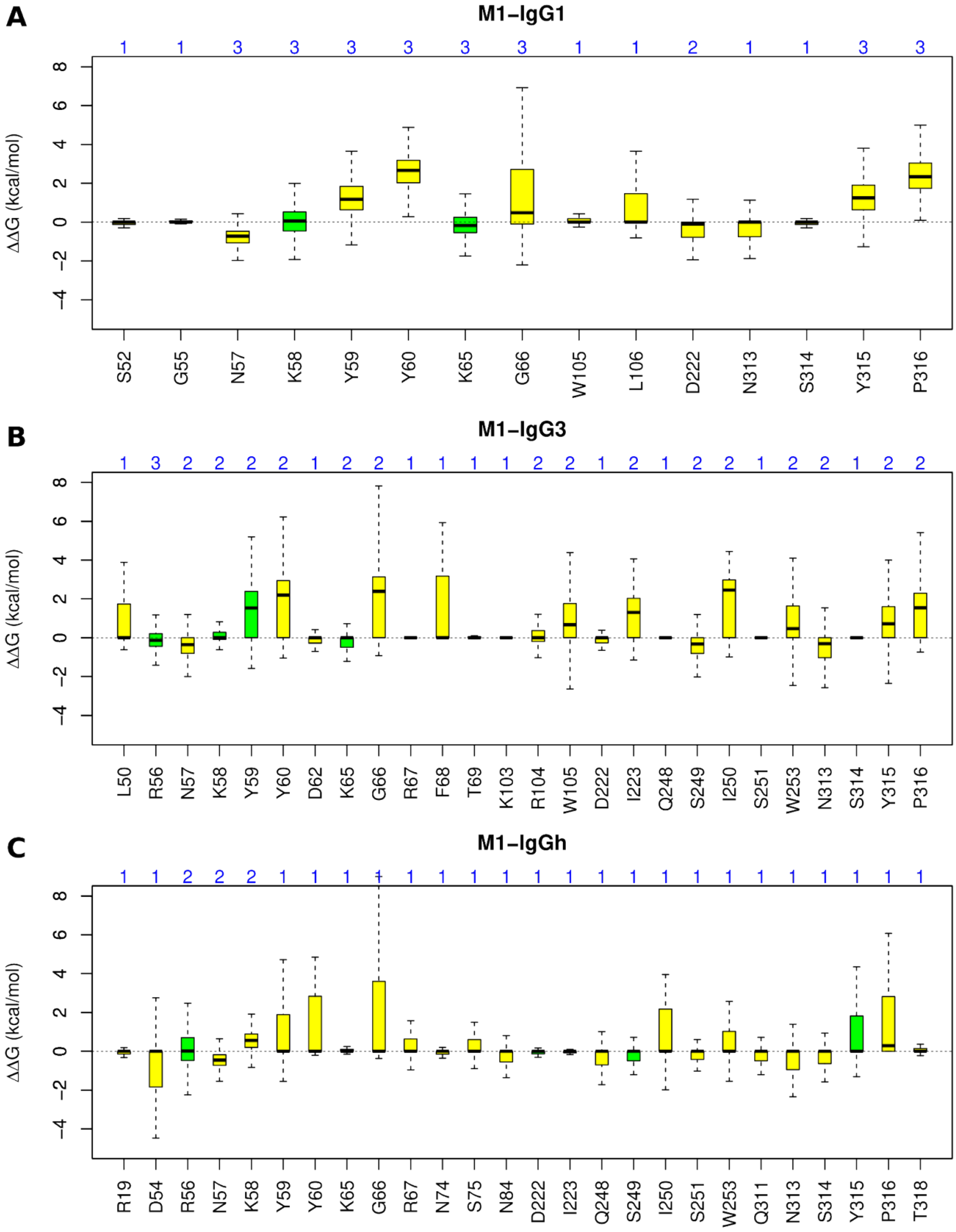

Supplemental Figure 9. Computational alanine scanning of the Fab2 residues at the interface with the M1. The results are reported for A) M1-IgG1, B) M1-IgG3, and C) M1-IgGh$_{62}$. The green boxes correspond to residues forming H-Bonds and salt bridges with the M1 protein. The blue nummer on top of each graph is the amount of replicates.

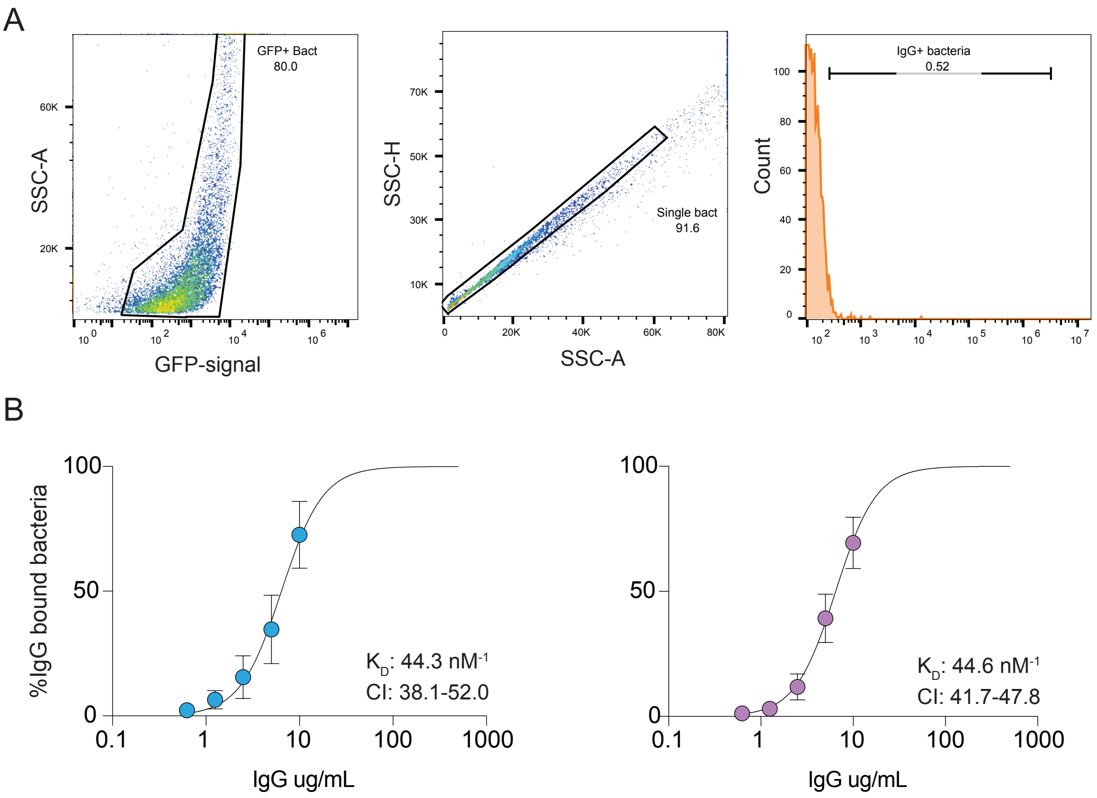

Supplementary Figure 10. Gating strategy and directly labeled affinity measurements. A Gating strategy for bacteria. Bacteria were gated for granularity (SSC-A) and GFP fluorescence (FITC-A). Thereafter single cell gate was done to exclude duplicate events based on granularity height (SSC-H) and area (SSC-A). To assess IgG binding to bacteria directly conjugated IgG with Alexa 647 (APC-A) and used unstained bacteria to set the gate. **B** Affinity curves for the directly conjugated antibodies. The data points are the mean value of four independent experiments with biological replicates. The KD values are shown with the 95% confidence interval indicated.

A

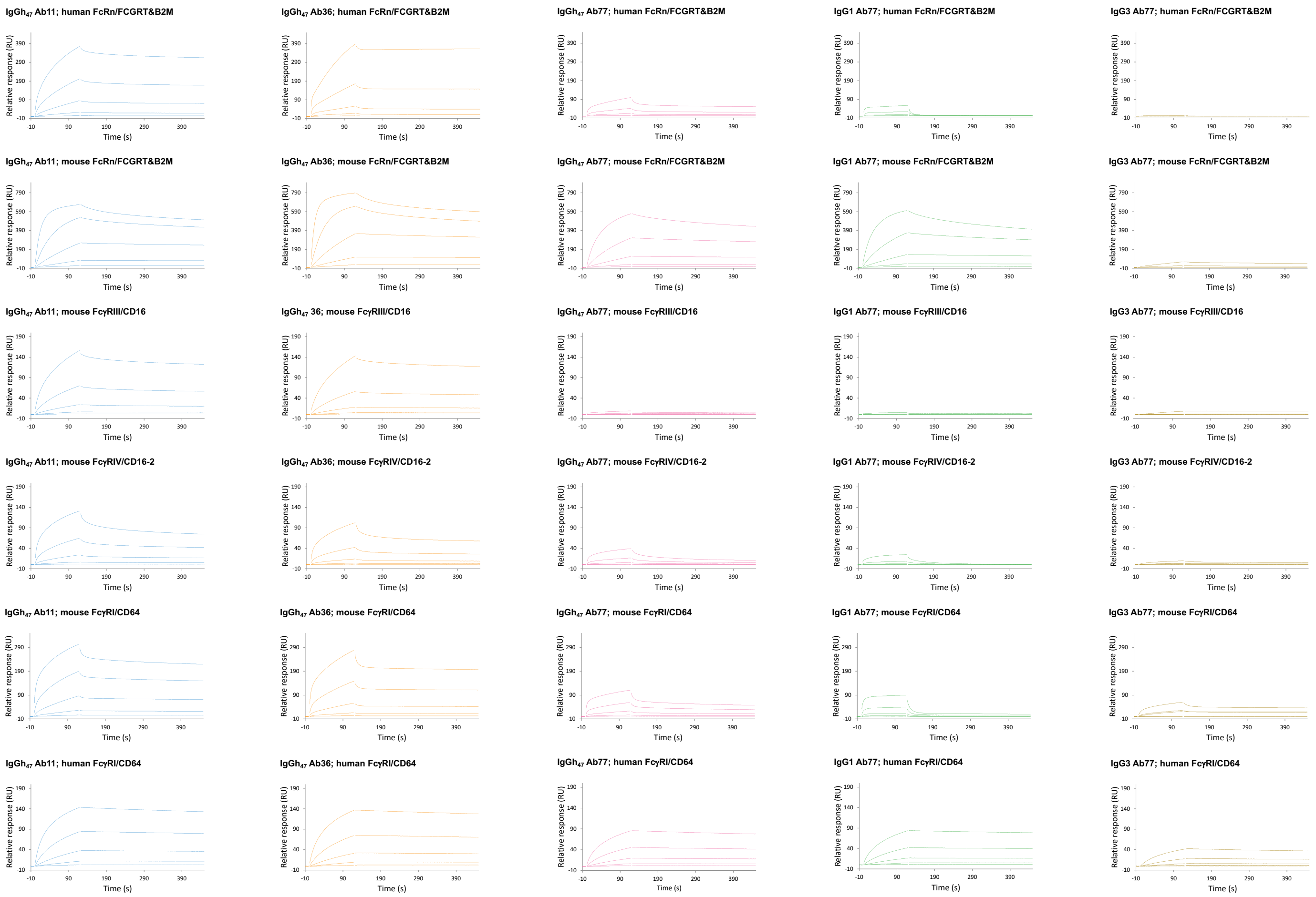

B

Receptor / kd	IgGh$_{47}$ Ab11 (s^{-1})	IgGh$_{47}$ Ab36 (s^{-1})	IgGh$_{47}$ Ab77 (s^{-1})	IgG1 Ab77 (s^{-1})	IgG3 Ab77 (s^{-1})
**human FcRn**	4.8×10^{-4}	2.5×10^{-4}	24×10^{-4}	880×10^{-4}	low binding
**mouse FcRn**	7.1×10^{-4}	8.5×10^{-4}	6.4×10^{-4}	10×10^{-4}	low binding
**mouse CD16**	7.4×10^{-4}	6.0×10^{-4}	low binding	low binding	low binding
**mouse CD16-2**	18×10^{-4}	20×10^{-4}	680×10^{-4}	275×10^{-4}	low binding
**mouse CD64**	8.3×10^{-4}	9.6×10^{-4}	30×10^{-4}	620×10^{-4}	14×10^{-4}
**human CD64**	1.7×10^{-4}	1.5×10^{-4}	2.3×10^{-4}	1.4×10^{-4}	3.3×10^{-4}

C

Receptor / KD	IgGh$_{47}$ Ab11 (M)	IgGh$_{47}$ Ab36 (M)	IgGh$_{47}$ Ab77 (M)	IgG1 Ab77 (M)	IgG3 Ab77 (M)
**human FcRn**	7.1×10^{-9}	6.6×10^{-9}	42×10^{-9}	300×10^{-9}	low binding
**mouse FcRn**	3.8×10^{-9}	3.5×10^{-9}	8.4×10^{-9}	9.9×10^{-9}	low binding
**mouse CD16**	15×10^{-9}	16×10^{-9}	low binding	low binding	low binding
**mouse CD16-2**	24×10^{-9}	36×10^{-9}	75×10^{-9}	415×10^{-9}	low binding
**mouse CD64**	7.0×10^{-9}	12×10^{-9}	28×10^{-9}	246×10^{-9}	14×10^{-9}
**human CD64**	2.1×10^{-9}	2.1×10^{-9}	3.5×10^{-9}	2.4×10^{-9}	8.1×10^{-9}

Supplemental Figure 11. Binding characteristics of human IgGh$_{47}$, IgG1, and IgG3 to Fc receptors as determined by surface plasmon resonance. A. Sensorgrams shown are (from left to right) binding profiles of SARS-CoV-2 spike protein-specific antibodies Ab11 IgGh$_{47}$, Ab36 IgGh$_{47}$, Ab77 IgGh$_{47}$, Ab77 IgG1, and Ab77IgG3 to (from top to bottom) human FcRn and mouse FcRn (both at pH 6.2), and mouse FcgRIII, mouse FcgRIV, mouse FcgRI, and human FcgRI. Binding to biotinylated FcR caught on streptavidin was assessed at 25°C at antibody concentrations of 400 nM, 100 nM, 25 nM, 6.2 nM, and 1.6 nM. **B-C**. Dissociation rate constants and affinity constants of the interactions.

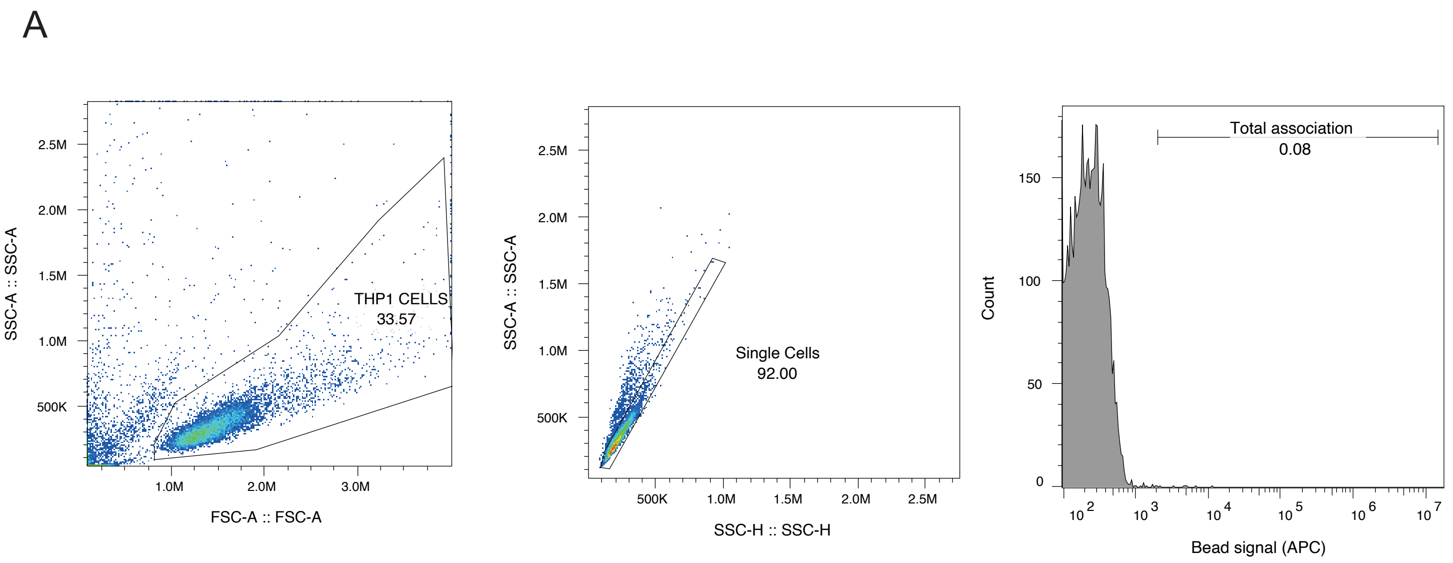

Supplemental Figure 12. Gating strategy for THP-1 cell phagocytosis of spike-beads.
A Shows gating strategy for THP-1 cells in flowcytometry. In short, cells only was used to gate cells for granularity and size, thereafter a single-cell gate was made to exclude duplicate events followed by a bead+ gate in the APC channel with cells only (bead-).

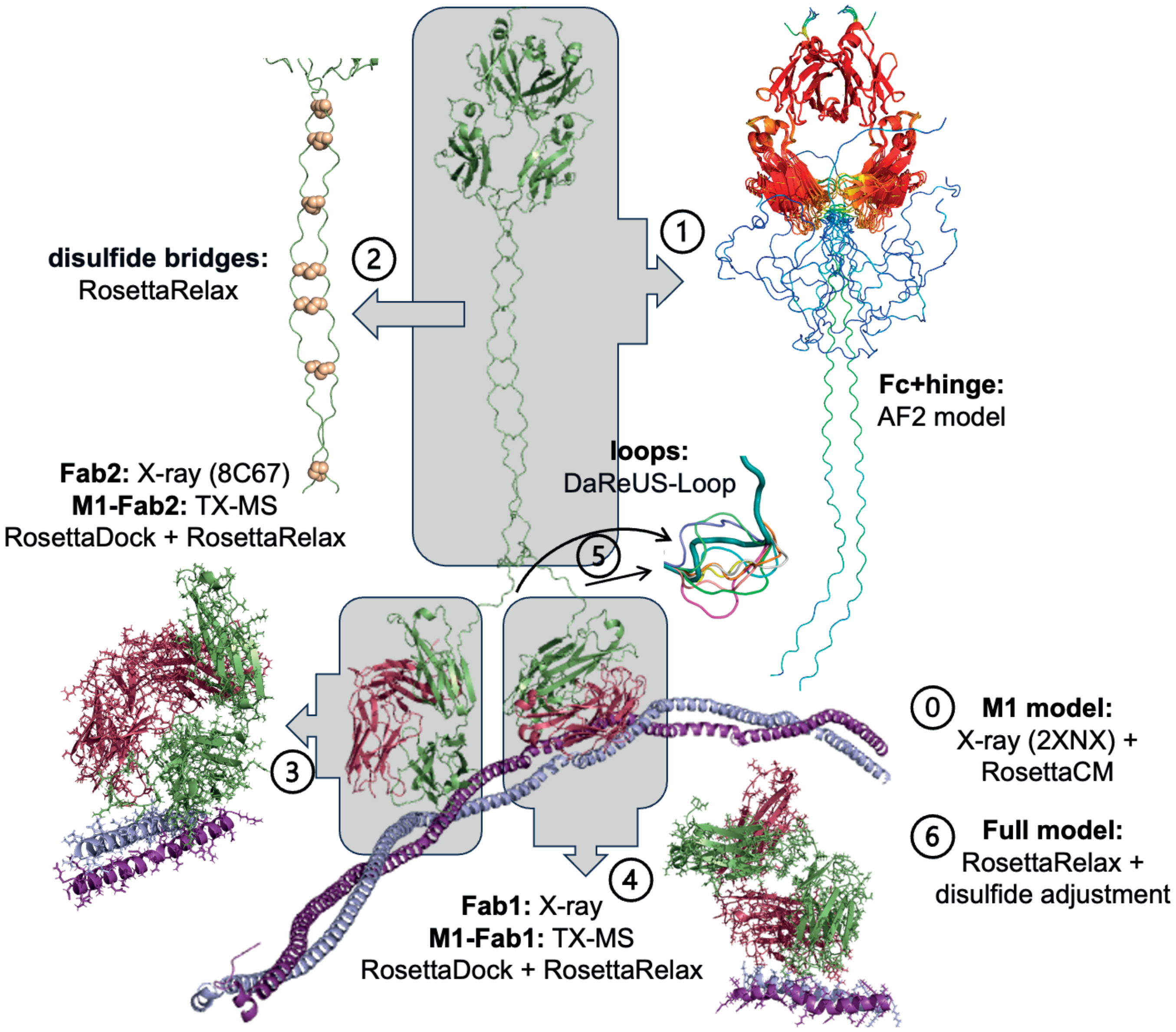

Supplemental Figure 13. Integrative structural modeling of M1-IgG3.
Step 0: the M1 model is used from our previous study where existing X-ray structure (2XNX-chains MN) were extended using Rosetta comparative modeling (RosettaCM) protocol. Step 1: the Fc domain was modeled with AlphaFold2. Top ranked models are aligned and colored based on pLDDT values (red means highly confident). Step 2: the model with elongated hinge region is selected and relaxed with Rosetta relax protocol with setting constraints on disulfide bridges. Step 3-4: the M1 fragments interactions with Fab domains of IgG were modeled based on cross-linking mass spectrometry data combined with RosettaDock models (TX-MS protocol). 4000 low-resolution docking models were generated to find the best matching pose with cross-linking constraints and then 100 high-resolution models were generated and sorted out based on the total Rosetta energy score. Step 5: loop regions connecting hinge to Fab domains were modeled with DaReUS-Loop. Step 6: the final model was relaxed once again, and disulfide-bridges were adjusted.

A

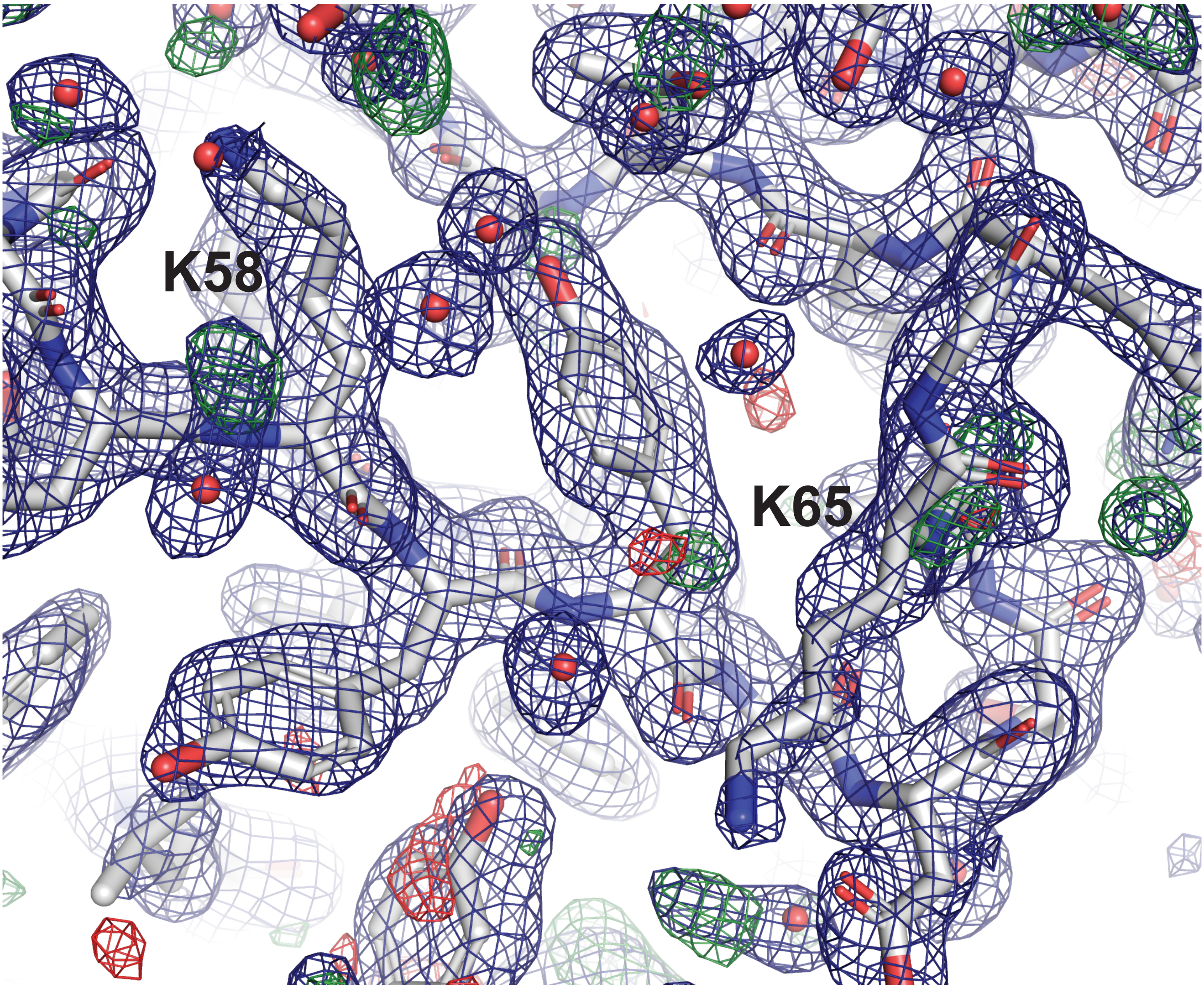

Supplementary Figure 14. Ab25 crystal structure

A The crystal structure of Ab25 showing residues K58 and K65 of chain C (see also Figure 3C, Fab2). The 2mFo-DFc electron density map contoured at 1.5 σ (blue), and the mFo-DFc contoured at 3.0 σ (green) and -3.0 σ (red).
