# Supplementary material for: The hinge-engineered IgG1-IgG3 hybrid subclass IgGh47 potently enhances Fc-mediated function of anti-streptococcal and SARS-CoV-2 antibodies
Source: Nat Commun. 2024 Apr 27;15:3600. doi: 10.1038/s41467-024-47928-8 (PMC11055898; doi:10.1038/s41467-024-47928-8)
Supplement: Supplementary file 3 — Description of Additional Supplementary Files [file 41467_2024_47928_MOESM3_ESM.pdf]

Description of Additional Supplementary Files

Supplementary Movie 1. Molecular dynamics simulation of IgG1-M1 repeat 1.

Supplementary Movie 2. Molecular dynamics simulation of IgG1-M1 repeat 2.

Supplementary Movie 3. Molecular dynamics simulation of IgG1-M1 repeat 3.

Supplementary Movie 4. Molecular dynamics simulation of IgG3-M1 repeat 1.

Supplementary Movie 5. Molecular dynamics simulation of IgG3-M1 repeat 2.

Supplementary Movie 6. Molecular dynamics simulation of IgG3-M1 repeat 3.

Supplementary Movie 7. Molecular dynamics simulation of IgGh-M1 repeat 1.

Supplementary Movie 8. Molecular dynamics simulation of IgGh-M1 repeat 2.

Supplementary Movie 9. Molecular dynamics simulation of IgGh-M1 repeat 3.
